# Bis(ferrocene­carboxyl­ato-κ*O*)bis­(2-pyridyl­methanol-κ^2^ *N*,*O*)cobalt(II)

**DOI:** 10.1107/S1600536810020805

**Published:** 2010-06-05

**Authors:** Youzhu Yu, Yuhua Guo, Daqi Wang, Dacheng Li

**Affiliations:** a Department of Chemistry and Environmental Engineering, Anyang Institute of Technology, Henan 455000, People’s Republic of China; b College of Chemistry and Chemical Engineering, Liaocheng University, Shandong 252059, People’s Republic of China

## Abstract

The title complex mol­ecule, [Fe_2_Co(C_5_H_5_)_2_(C_6_H_4_O_2_)_2_(C_6_H_7_NO)_2_], has a crystallographic imposed centre of symmetry. The Co^II^ atom displays a distorted octa­hedral coordination geometry, provided by the O atoms of two monodentate ferrocene­carboxyl­ate anions and by the N and O atoms of two 2-pyridyl­methanol mol­ecule. The mol­ecular conformation is stabilized by intra­molecular C—H⋯O hydrogen bonds.

## Related literature

For related structures, see: Salazar-Mendoza *et al.* (2007 ▶); Meng *et al.* (2004 ▶).
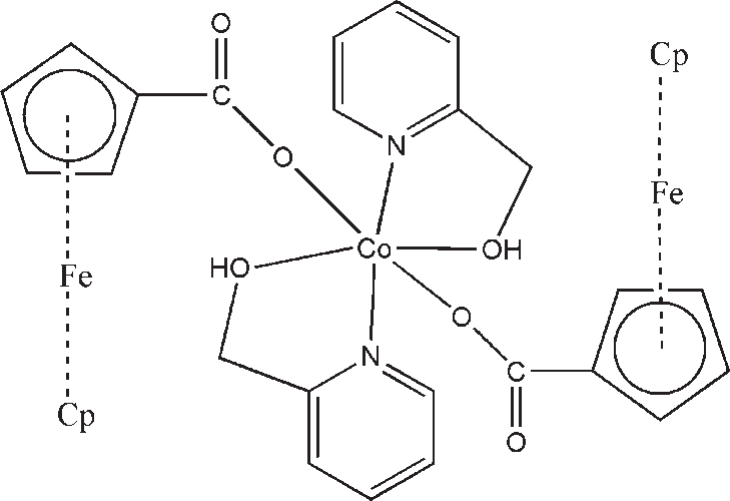


## Experimental

### 

#### Crystal data

[Fe_2_Co(C_5_H_5_)_2_(C_6_H_4_O_2_)_2_(C_6_H_7_NO)_2_]
*M* *_r_* = 735.25
Monoclinic, 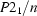

*a* = 12.5790 (12) Å
*b* = 7.7905 (9) Å
*c* = 15.2975 (15) Å
β = 90.141 (1)°
*V* = 1499.1 (3) Å^3^
*Z* = 2
Mo *K*α radiation
μ = 1.55 mm^−1^
*T* = 298 K
0.40 × 0.37 × 0.28 mm

#### Data collection

Bruker SMART 1000 CCD area-detector diffractometer
Absorption correction: multi-scan (*SADABS*; Sheldrick, 1996 ▶) *T*_min_ = 0.576, *T*_max_ = 0.670
7475 measured reflections
2642 independent reflections
1651 reflections with *I* > 2σ(*I*)
*R*_int_ = 0.045

#### Refinement

*R*[*F* ^2^ > 2σ(*F* ^2^)] = 0.042
*wR*(*F* ^2^) = 0.125
*S* = 1.01
2642 reflections
205 parameters
H-atom parameters constrained
Δρ_max_ = 0.50 e Å^−3^
Δρ_min_ = −0.30 e Å^−3^

### 

Data collection: *SMART* (Siemens, 1996 ▶); cell refinement: *SAINT* (Siemens, 1996 ▶); data reduction: *SAINT*; program(s) used to solve structure: *SHELXS97* (Sheldrick, 2008 ▶); program(s) used to refine structure: *SHELXL97* (Sheldrick, 2008 ▶); molecular graphics: *SHELXTL* (Sheldrick, 2008 ▶); software used to prepare material for publication: *SHELXTL*.

## Supplementary Material

Crystal structure: contains datablocks I, global. DOI: 10.1107/S1600536810020805/rz2457sup1.cif

Structure factors: contains datablocks I. DOI: 10.1107/S1600536810020805/rz2457Isup2.hkl

Additional supplementary materials:  crystallographic information; 3D view; checkCIF report

## Figures and Tables

**Table 1:** Hydrogen-bond geometry (Å, °)

*D*—H⋯*A*	*D*—H	H⋯*A*	*D*⋯*A*	*D*—H⋯*A*
O6—H6⋯O4	0.82	1.70	2.516 (5)	176

**Fig. 1.**
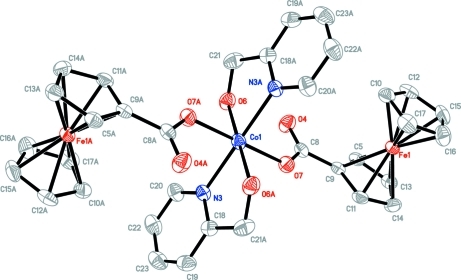
The molecular structure of the title compound, with atom labels and 30% probability displacement ellipsoids. Atoms labelled wit suffix A are generated by the symmetry operation -x, -y, -z. Hydrogen atoms are omitted for clarity.

## supplementary crystallographic information

### Comment

Ferrocene is an interesting redox centre for the construction of molecular architectures presenting magnetic, optical or electrochemical properties. As a contribution to this field, we report here the synthesis and structure of the title compound.

The title compound possesses crystallographic imposed centre of symmetry (Fig. 1). The cobalt(II) atom is six-coordinated in a distorted octahedral geometry by two O atoms of two monodentate ferrocenecarboxylare anions, two N and two O atoms of two pyridinemethanol molecules. Bond lengths and angles involving the metal centre are typical and comparable with those observed in related cobalt(II) complexes (Salazar-Mendoza *et al.*, 2007; Meng *et al.*, 2004). The conformation of the complex molecule is stabilized by intramolecular C—H···O hydrogen bonds (Table 1).

### Experimental

A methanol solution of 2-pyridinemethanol (0.4 mmol, 3 ml) was added into a 10 ml methanol solution of cobalt(II) dichloride (0.2 mmol, 47.6 mg) at 293 K, then 10 ml of a methanol solution of ferrocenecarboxyl sodium (0.4 mmol, 100.8 mg) was added dropwise and the mixture stirred for 6 h. The resulting orange solution was allowed to stand at room temperature for about one week, whereupon red block crystals suitable for X-ray diffraction analysis were obtained.

### Refinement

All H atoms were placed in geometrically idealized positions and treated as riding on their parent atoms, with C—H = 0.93-0.98 Å, O—H = 0.82 Å, and with *U*_iso_(H) = 1.2*U*_eq_(C) or 1.5*U*_eq_(O).

### Crystal data

**Table:**

[Fe_2_Co(C_5_H_5_)_2_(C_6_H_4_O_2_)_2_(C_6_H_7_NO)_2_]	*F*(000) = 754
*M**_r_* = 735.25	*D*_x_ = 1.629 Mg m^−^^3^
Monoclinic, *P*2_1_/*n*	Mo *K*α radiation, λ = 0.71073 Å
Hall symbol: -P 2yn	Cell parameters from 1624 reflections
*a* = 12.5790 (12) Å	θ = 2.9–26.5°
*b* = 7.7905 (9) Å	µ = 1.55 mm^−^^1^
*c* = 15.2975 (15) Å	*T* = 298 K
β = 90.141 (1)°	Block, red
*V* = 1499.1 (3) Å^3^	0.40 × 0.37 × 0.28 mm
*Z* = 2	

### Data collection

**Table:**

Bruker SMART 1000 CCD area-detector diffractometer	2642 independent reflections
Radiation source: fine-focus sealed tube	1651 reflections with *I* > 2σ(*I*)
graphite	*R*_int_ = 0.045
phi and ω scans	θ_max_ = 25.0°, θ_min_ = 2.1°
Absorption correction: multi-scan (*SADABS*; Sheldrick, 1996)	*h* = −14→14
*T*_min_ = 0.576, *T*_max_ = 0.670	*k* = −9→9
7475 measured reflections	*l* = −13→18

### Refinement

**Table:**

Refinement on *F*^2^	Primary atom site location: structure-invariant direct methods
Least-squares matrix: full	Secondary atom site location: difference Fourier map
*R*[*F*^2^ > 2σ(*F*^2^)] = 0.042	Hydrogen site location: inferred from neighbouring sites
*wR*(*F*^2^) = 0.125	H-atom parameters constrained
*S* = 1.01	*w* = 1/[σ^2^(*F*_o_^2^) + (0.0581*P*)^2^ + 0.7962*P*] where *P* = (*F*_o_^2^ + 2*F*_c_^2^)/3
2642 reflections	(Δ/σ)_max_ < 0.001
205 parameters	Δρ_max_ = 0.50 e Å^−^^3^
0 restraints	Δρ_min_ = −0.30 e Å^−^^3^

### Fractional atomic coordinates and isotropic or equivalent isotropic displacement parameters (Å^2^)

**Table:**

	*x*	*y*	*z*	*U*_iso_*/*U*_eq_	
Co1	0.0000	0.0000	0.0000	0.0433 (3)	
Fe1	0.10119 (5)	−0.12834 (9)	0.35046 (4)	0.0398 (2)	
N3	0.1164 (3)	0.1549 (5)	−0.0649 (2)	0.0447 (10)	
O4	−0.0149 (3)	0.1926 (5)	0.1934 (2)	0.0590 (10)	
C5	0.1235 (3)	0.1298 (6)	0.3445 (3)	0.0413 (11)	
H5	0.0729	0.2187	0.3625	0.050*	
O6	−0.1077 (3)	0.1896 (4)	0.0475 (2)	0.0546 (9)	
H6	−0.0750	0.1887	0.0939	0.082*	
O7	0.0864 (2)	0.0167 (4)	0.1132 (2)	0.0506 (9)	
C8	0.0616 (4)	0.0908 (6)	0.1841 (3)	0.0442 (12)	
C9	0.1318 (4)	0.0544 (6)	0.2604 (3)	0.0400 (11)	
C10	−0.0427 (4)	−0.2295 (7)	0.3206 (3)	0.0529 (13)	
H10	−0.0913	−0.1887	0.2749	0.063*	
C11	0.2153 (3)	−0.0659 (7)	0.2616 (3)	0.0479 (13)	
H11	0.2382	−0.1371	0.2123	0.057*	
C12	−0.0401 (4)	−0.1730 (7)	0.4084 (3)	0.0567 (15)	
H12	−0.0867	−0.0859	0.4342	0.068*	
C13	0.2033 (4)	0.0544 (7)	0.3968 (3)	0.0506 (13)	
H13	0.2165	0.0800	0.4586	0.061*	
C14	0.2592 (4)	−0.0674 (7)	0.3461 (3)	0.0549 (14)	
H14	0.3181	−0.1398	0.3663	0.066*	
C15	0.0390 (5)	−0.2629 (8)	0.4519 (3)	0.0628 (15)	
H15	0.0584	−0.2494	0.5136	0.075*	
C16	0.0875 (5)	−0.3736 (7)	0.3926 (4)	0.0669 (16)	
H16	0.1466	−0.4516	0.4056	0.080*	
C17	0.0368 (4)	−0.3548 (7)	0.3105 (4)	0.0600 (15)	
H17	0.0539	−0.4172	0.2567	0.072*	
C19	0.2825 (4)	0.1471 (8)	−0.1388 (3)	0.0591 (15)	
H19	0.3420	0.0851	−0.1561	0.071*	
C18	0.2036 (4)	0.0693 (7)	−0.0893 (3)	0.0470 (12)	
C22	0.1832 (5)	0.4044 (8)	−0.1359 (3)	0.0652 (16)	
H22	0.1746	0.5195	−0.1505	0.078*	
C20	0.1064 (4)	0.3190 (7)	−0.0874 (3)	0.0562 (14)	
H20	0.0460	0.3786	−0.0701	0.067*	
C23	0.2716 (5)	0.3163 (8)	−0.1619 (4)	0.0665 (16)	
H23	0.3238	0.3704	−0.1949	0.080*	
C21	−0.2095 (4)	0.1131 (7)	0.0597 (4)	0.0672 (16)	
H21A	−0.2486	0.1180	0.0050	0.081*	
H21B	−0.2487	0.1792	0.1026	0.081*	

### Atomic displacement parameters (Å^2^)

**Table:**

	*U*^11^	*U*^22^	*U*^33^	*U*^12^	*U*^13^	*U*^23^
Co1	0.0505 (6)	0.0436 (6)	0.0359 (5)	0.0067 (5)	0.0074 (4)	0.0007 (4)
Fe1	0.0420 (4)	0.0437 (4)	0.0338 (4)	−0.0004 (3)	0.0057 (3)	−0.0016 (3)
N3	0.050 (2)	0.045 (3)	0.039 (2)	0.000 (2)	0.0047 (18)	0.0012 (19)
O4	0.060 (2)	0.064 (2)	0.053 (2)	0.019 (2)	−0.0047 (17)	−0.0092 (18)
C5	0.042 (3)	0.038 (3)	0.043 (3)	−0.009 (2)	0.008 (2)	−0.007 (2)
O6	0.057 (2)	0.050 (2)	0.056 (2)	0.0128 (17)	−0.0021 (17)	0.0002 (17)
O7	0.054 (2)	0.062 (2)	0.0353 (19)	0.0065 (18)	0.0044 (15)	−0.0044 (17)
C8	0.048 (3)	0.041 (3)	0.044 (3)	−0.008 (3)	0.007 (2)	0.006 (2)
C9	0.037 (3)	0.044 (3)	0.039 (3)	−0.004 (2)	0.010 (2)	0.001 (2)
C10	0.046 (3)	0.056 (3)	0.056 (3)	−0.012 (3)	0.001 (2)	0.004 (3)
C11	0.039 (3)	0.064 (3)	0.040 (3)	−0.003 (2)	0.013 (2)	−0.003 (2)
C12	0.052 (3)	0.063 (4)	0.055 (4)	−0.017 (3)	0.024 (3)	−0.006 (3)
C13	0.051 (3)	0.066 (4)	0.036 (3)	−0.014 (3)	0.001 (2)	−0.008 (2)
C14	0.038 (3)	0.078 (4)	0.049 (3)	0.002 (3)	0.000 (2)	−0.003 (3)
C15	0.081 (4)	0.067 (4)	0.040 (3)	−0.023 (3)	0.000 (3)	0.015 (3)
C16	0.087 (4)	0.044 (3)	0.069 (4)	0.003 (3)	0.003 (3)	0.009 (3)
C17	0.071 (4)	0.050 (3)	0.060 (4)	−0.013 (3)	0.005 (3)	−0.011 (3)
C19	0.046 (3)	0.073 (4)	0.059 (4)	−0.008 (3)	0.002 (3)	−0.013 (3)
C18	0.046 (3)	0.048 (3)	0.047 (3)	−0.001 (3)	0.001 (2)	−0.007 (2)
C22	0.089 (5)	0.053 (4)	0.053 (4)	−0.018 (3)	0.004 (3)	−0.001 (3)
C20	0.071 (4)	0.051 (3)	0.046 (3)	0.002 (3)	0.010 (3)	−0.005 (3)
C23	0.070 (4)	0.075 (5)	0.054 (4)	−0.029 (3)	0.008 (3)	−0.010 (3)
C21	0.049 (3)	0.068 (4)	0.085 (4)	0.013 (3)	0.001 (3)	−0.004 (3)

### Geometric parameters (Å, °)

**Table:**

Co1—O7^i^	2.046 (3)	C10—C17	1.406 (7)
Co1—O7	2.046 (3)	C10—C12	1.413 (7)
Co1—O6^i^	2.133 (3)	C10—H10	0.9800
Co1—O6	2.133 (3)	C11—C14	1.405 (6)
Co1—N3^i^	2.142 (4)	C11—H11	0.9800
Co1—N3	2.142 (4)	C12—C15	1.386 (7)
Fe1—C12	2.018 (5)	C12—H12	0.9800
Fe1—C9	2.019 (5)	C13—C14	1.415 (7)
Fe1—C16	2.024 (5)	C13—H13	0.9800
Fe1—C10	2.026 (5)	C14—H14	0.9800
Fe1—C15	2.031 (5)	C15—C16	1.393 (7)
Fe1—C5	2.033 (5)	C15—H15	0.9800
Fe1—C17	2.034 (5)	C16—C17	1.414 (7)
Fe1—C11	2.038 (4)	C16—H16	0.9800
Fe1—C13	2.043 (5)	C17—H17	0.9800
Fe1—C14	2.045 (5)	C19—C23	1.371 (8)
N3—C20	1.330 (6)	C19—C18	1.388 (7)
N3—C18	1.338 (6)	C19—H19	0.9300
O4—C8	1.255 (5)	C18—C21^i^	1.493 (7)
C5—C13	1.411 (6)	C22—C23	1.367 (8)
C5—C9	1.418 (6)	C22—C20	1.389 (7)
C5—H5	0.9800	C22—H22	0.9300
O6—C21	1.424 (6)	C20—H20	0.9300
O6—H6	0.8200	C23—H23	0.9300
O7—C8	1.269 (5)	C21—C18^i^	1.493 (7)
C8—C9	1.488 (6)	C21—H21A	0.9700
C9—C11	1.408 (6)	C21—H21B	0.9700
			
O7^i^—Co1—O7	180.0 (2)	C11—C9—C5	108.7 (4)
O7^i^—Co1—O6^i^	90.26 (12)	C11—C9—C8	125.3 (4)
O7—Co1—O6^i^	89.74 (12)	C5—C9—C8	126.0 (4)
O7^i^—Co1—O6	89.74 (12)	C11—C9—Fe1	70.4 (3)
O7—Co1—O6	90.26 (12)	C5—C9—Fe1	70.0 (3)
O6^i^—Co1—O6	180.0 (2)	C8—C9—Fe1	123.7 (3)
O7^i^—Co1—N3^i^	89.65 (14)	C17—C10—C12	107.8 (5)
O7—Co1—N3^i^	90.35 (14)	C17—C10—Fe1	70.1 (3)
O6^i^—Co1—N3^i^	101.73 (14)	C12—C10—Fe1	69.3 (3)
O6—Co1—N3^i^	78.27 (14)	C17—C10—H10	126.1
O7^i^—Co1—N3	90.35 (14)	C12—C10—H10	126.1
O7—Co1—N3	89.65 (14)	Fe1—C10—H10	126.1
O6^i^—Co1—N3	78.27 (14)	C14—C11—C9	108.0 (4)
O6—Co1—N3	101.73 (14)	C14—C11—Fe1	70.2 (3)
N3^i^—Co1—N3	180.0 (3)	C9—C11—Fe1	69.0 (3)
C12—Fe1—C9	126.2 (2)	C14—C11—H11	126.0
C12—Fe1—C16	67.8 (2)	C9—C11—H11	126.0
C9—Fe1—C16	154.1 (2)	Fe1—C11—H11	126.0
C12—Fe1—C10	40.91 (19)	C15—C12—C10	108.3 (5)
C9—Fe1—C10	107.01 (19)	C15—C12—Fe1	70.5 (3)
C16—Fe1—C10	68.1 (2)	C10—C12—Fe1	69.8 (3)
C12—Fe1—C15	40.1 (2)	C15—C12—H12	125.9
C9—Fe1—C15	163.7 (2)	C10—C12—H12	125.9
C16—Fe1—C15	40.2 (2)	Fe1—C12—H12	125.9
C10—Fe1—C15	68.0 (2)	C5—C13—C14	108.8 (4)
C12—Fe1—C5	108.2 (2)	C5—C13—Fe1	69.4 (3)
C9—Fe1—C5	40.98 (17)	C14—C13—Fe1	69.8 (3)
C16—Fe1—C5	163.8 (2)	C5—C13—H13	125.6
C10—Fe1—C5	119.9 (2)	C14—C13—H13	125.6
C15—Fe1—C5	126.8 (2)	Fe1—C13—H13	125.6
C12—Fe1—C17	68.4 (2)	C11—C14—C13	107.7 (4)
C9—Fe1—C17	118.9 (2)	C11—C14—Fe1	69.6 (3)
C16—Fe1—C17	40.8 (2)	C13—C14—Fe1	69.7 (3)
C10—Fe1—C17	40.5 (2)	C11—C14—H14	126.2
C15—Fe1—C17	68.2 (2)	C13—C14—H14	126.2
C5—Fe1—C17	154.1 (2)	Fe1—C14—H14	126.2
C12—Fe1—C11	163.0 (2)	C12—C15—C16	108.4 (5)
C9—Fe1—C11	40.62 (18)	C12—C15—Fe1	69.5 (3)
C16—Fe1—C11	119.9 (2)	C16—C15—Fe1	69.6 (3)
C10—Fe1—C11	125.0 (2)	C12—C15—H15	125.8
C15—Fe1—C11	154.9 (2)	C16—C15—H15	125.8
C5—Fe1—C11	68.70 (19)	Fe1—C15—H15	125.8
C17—Fe1—C11	106.7 (2)	C15—C16—C17	108.4 (5)
C12—Fe1—C13	121.4 (2)	C15—C16—Fe1	70.2 (3)
C9—Fe1—C13	67.95 (19)	C17—C16—Fe1	70.0 (3)
C16—Fe1—C13	127.0 (2)	C15—C16—H16	125.8
C10—Fe1—C13	155.5 (2)	C17—C16—H16	125.8
C15—Fe1—C13	109.7 (2)	Fe1—C16—H16	125.8
C5—Fe1—C13	40.49 (18)	C10—C17—C16	107.1 (5)
C17—Fe1—C13	163.3 (2)	C10—C17—Fe1	69.4 (3)
C11—Fe1—C13	67.80 (19)	C16—C17—Fe1	69.2 (3)
C12—Fe1—C14	155.7 (2)	C10—C17—H17	126.5
C9—Fe1—C14	68.1 (2)	C16—C17—H17	126.5
C16—Fe1—C14	108.2 (2)	Fe1—C17—H17	126.5
C10—Fe1—C14	162.0 (2)	C23—C19—C18	119.3 (5)
C15—Fe1—C14	121.4 (2)	C23—C19—H19	120.4
C5—Fe1—C14	68.6 (2)	C18—C19—H19	120.4
C17—Fe1—C14	125.3 (2)	N3—C18—C19	121.5 (5)
C11—Fe1—C14	40.24 (18)	N3—C18—C21^i^	115.4 (5)
C13—Fe1—C14	40.5 (2)	C19—C18—C21^i^	123.1 (5)
C20—N3—C18	118.9 (4)	C23—C22—C20	118.8 (6)
C20—N3—Co1	126.7 (3)	C23—C22—H22	120.6
C18—N3—Co1	114.2 (3)	C20—C22—H22	120.6
C13—C5—C9	106.7 (4)	N3—C20—C22	122.3 (5)
C13—C5—Fe1	70.2 (3)	N3—C20—H20	118.9
C9—C5—Fe1	69.0 (3)	C22—C20—H20	118.9
C13—C5—H5	126.6	C22—C23—C19	119.2 (5)
C9—C5—H5	126.6	C22—C23—H23	120.4
Fe1—C5—H5	126.6	C19—C23—H23	120.4
C21—O6—Co1	109.1 (3)	O6—C21—C18^i^	113.2 (4)
C21—O6—H6	109.5	O6—C21—H21A	108.9
Co1—O6—H6	88.3	C18^i^—C21—H21A	108.9
C8—O7—Co1	128.4 (3)	O6—C21—H21B	108.9
O4—C8—O7	125.0 (4)	C18^i^—C21—H21B	108.9
O4—C8—C9	119.0 (4)	H21A—C21—H21B	107.8
O7—C8—C9	116.0 (4)		
			
O7^i^—Co1—N3—C20	74.7 (4)	C15—Fe1—C12—C10	−119.0 (5)
O7—Co1—N3—C20	−105.3 (4)	C5—Fe1—C12—C10	115.0 (3)
O6^i^—Co1—N3—C20	164.9 (4)	C17—Fe1—C12—C10	−37.7 (3)
O6—Co1—N3—C20	−15.1 (4)	C11—Fe1—C12—C10	38.5 (9)
O7^i^—Co1—N3—C18	−100.3 (3)	C13—Fe1—C12—C10	157.4 (3)
O7—Co1—N3—C18	79.7 (3)	C14—Fe1—C12—C10	−167.0 (5)
O6^i^—Co1—N3—C18	−10.1 (3)	C9—C5—C13—C14	−0.7 (5)
O6—Co1—N3—C18	169.9 (3)	Fe1—C5—C13—C14	58.8 (4)
C12—Fe1—C5—C13	117.4 (3)	C9—C5—C13—Fe1	−59.5 (3)
C9—Fe1—C5—C13	−117.8 (4)	C12—Fe1—C13—C5	−81.2 (3)
C16—Fe1—C5—C13	44.6 (9)	C9—Fe1—C13—C5	38.7 (3)
C10—Fe1—C5—C13	160.6 (3)	C16—Fe1—C13—C5	−165.8 (3)
C15—Fe1—C5—C13	76.9 (4)	C10—Fe1—C13—C5	−43.9 (6)
C17—Fe1—C5—C13	−164.7 (4)	C15—Fe1—C13—C5	−124.0 (3)
C11—Fe1—C5—C13	−80.3 (3)	C17—Fe1—C13—C5	156.4 (6)
C14—Fe1—C5—C13	−37.0 (3)	C11—Fe1—C13—C5	82.7 (3)
C12—Fe1—C5—C9	−124.8 (3)	C14—Fe1—C13—C5	120.4 (4)
C16—Fe1—C5—C9	162.5 (7)	C12—Fe1—C13—C14	158.4 (3)
C10—Fe1—C5—C9	−81.6 (3)	C9—Fe1—C13—C14	−81.7 (3)
C15—Fe1—C5—C9	−165.3 (3)	C16—Fe1—C13—C14	73.8 (4)
C17—Fe1—C5—C9	−46.9 (6)	C10—Fe1—C13—C14	−164.3 (4)
C11—Fe1—C5—C9	37.5 (3)	C15—Fe1—C13—C14	115.6 (3)
C13—Fe1—C5—C9	117.8 (4)	C5—Fe1—C13—C14	−120.4 (4)
C14—Fe1—C5—C9	80.8 (3)	C17—Fe1—C13—C14	36.0 (8)
O7^i^—Co1—O6—C21	65.6 (3)	C11—Fe1—C13—C14	−37.7 (3)
O7—Co1—O6—C21	−114.4 (3)	C9—C11—C14—C13	−0.8 (5)
N3—Co1—O6—C21	155.9 (3)	Fe1—C11—C14—C13	−59.5 (3)
O6^i^—Co1—O7—C8	−168.1 (4)	C9—C11—C14—Fe1	58.8 (3)
O6—Co1—O7—C8	11.9 (4)	C5—C13—C14—C11	0.9 (6)
N3^i^—Co1—O7—C8	−66.4 (4)	Fe1—C13—C14—C11	59.5 (3)
N3—Co1—O7—C8	113.6 (4)	C5—C13—C14—Fe1	−58.5 (3)
Co1—O7—C8—O4	−12.2 (7)	C12—Fe1—C14—C11	−168.8 (5)
Co1—O7—C8—C9	169.2 (3)	C9—Fe1—C14—C11	−37.7 (3)
C13—C5—C9—C11	0.3 (5)	C16—Fe1—C14—C11	115.0 (3)
Fe1—C5—C9—C11	−60.0 (3)	C10—Fe1—C14—C11	39.8 (8)
C13—C5—C9—C8	178.1 (4)	C15—Fe1—C14—C11	157.2 (3)
Fe1—C5—C9—C8	117.8 (5)	C5—Fe1—C14—C11	−81.9 (3)
C13—C5—C9—Fe1	60.3 (3)	C17—Fe1—C14—C11	73.0 (4)
O4—C8—C9—C11	175.0 (4)	C13—Fe1—C14—C11	−118.9 (4)
O7—C8—C9—C11	−6.4 (7)	C12—Fe1—C14—C13	−49.9 (7)
O4—C8—C9—C5	−2.5 (7)	C9—Fe1—C14—C13	81.2 (3)
O7—C8—C9—C5	176.1 (4)	C16—Fe1—C14—C13	−126.1 (3)
O4—C8—C9—Fe1	86.2 (5)	C10—Fe1—C14—C13	158.7 (6)
O7—C8—C9—Fe1	−95.2 (5)	C15—Fe1—C14—C13	−83.9 (4)
C12—Fe1—C9—C11	−165.3 (3)	C5—Fe1—C14—C13	37.0 (3)
C16—Fe1—C9—C11	−49.5 (6)	C17—Fe1—C14—C13	−168.1 (3)
C10—Fe1—C9—C11	−124.3 (3)	C11—Fe1—C14—C13	118.9 (4)
C15—Fe1—C9—C11	166.1 (7)	C10—C12—C15—C16	0.9 (6)
C5—Fe1—C9—C11	119.4 (4)	Fe1—C12—C15—C16	−59.0 (4)
C17—Fe1—C9—C11	−82.0 (3)	C10—C12—C15—Fe1	59.8 (3)
C13—Fe1—C9—C11	81.1 (3)	C9—Fe1—C15—C12	37.0 (9)
C14—Fe1—C9—C11	37.3 (3)	C16—Fe1—C15—C12	−119.9 (5)
C12—Fe1—C9—C5	75.3 (3)	C10—Fe1—C15—C12	−38.1 (3)
C16—Fe1—C9—C5	−168.9 (5)	C5—Fe1—C15—C12	73.5 (4)
C10—Fe1—C9—C5	116.2 (3)	C17—Fe1—C15—C12	−82.0 (3)
C15—Fe1—C9—C5	46.6 (8)	C11—Fe1—C15—C12	−164.7 (4)
C17—Fe1—C9—C5	158.6 (3)	C13—Fe1—C15—C12	115.7 (3)
C11—Fe1—C9—C5	−119.4 (4)	C14—Fe1—C15—C12	159.0 (3)
C13—Fe1—C9—C5	−38.3 (3)	C12—Fe1—C15—C16	119.9 (5)
C14—Fe1—C9—C5	−82.1 (3)	C9—Fe1—C15—C16	156.8 (7)
C12—Fe1—C9—C8	−45.3 (5)	C10—Fe1—C15—C16	81.7 (4)
C16—Fe1—C9—C8	70.5 (7)	C5—Fe1—C15—C16	−166.6 (3)
C10—Fe1—C9—C8	−4.3 (4)	C17—Fe1—C15—C16	37.9 (3)
C15—Fe1—C9—C8	−73.9 (9)	C11—Fe1—C15—C16	−44.9 (6)
C5—Fe1—C9—C8	−120.6 (5)	C13—Fe1—C15—C16	−124.4 (4)
C17—Fe1—C9—C8	38.0 (5)	C14—Fe1—C15—C16	−81.1 (4)
C11—Fe1—C9—C8	120.0 (5)	C12—C15—C16—C17	−0.9 (6)
C13—Fe1—C9—C8	−158.9 (4)	Fe1—C15—C16—C17	−59.7 (4)
C14—Fe1—C9—C8	157.3 (5)	C12—C15—C16—Fe1	58.9 (4)
C12—Fe1—C10—C17	−119.0 (5)	C12—Fe1—C16—C15	−37.1 (3)
C9—Fe1—C10—C17	114.8 (3)	C9—Fe1—C16—C15	−165.4 (4)
C16—Fe1—C10—C17	−38.2 (3)	C10—Fe1—C16—C15	−81.4 (4)
C15—Fe1—C10—C17	−81.7 (4)	C5—Fe1—C16—C15	41.5 (9)
C5—Fe1—C10—C17	157.5 (3)	C17—Fe1—C16—C15	−119.3 (5)
C11—Fe1—C10—C17	73.8 (4)	C11—Fe1—C16—C15	159.8 (3)
C13—Fe1—C10—C17	−171.2 (4)	C13—Fe1—C16—C15	76.3 (4)
C14—Fe1—C10—C17	43.5 (8)	C14—Fe1—C16—C15	117.4 (4)
C9—Fe1—C10—C12	−126.2 (3)	C12—Fe1—C16—C17	82.2 (4)
C16—Fe1—C10—C12	80.9 (4)	C9—Fe1—C16—C17	−46.1 (6)
C15—Fe1—C10—C12	37.4 (3)	C10—Fe1—C16—C17	37.9 (3)
C5—Fe1—C10—C12	−83.4 (4)	C15—Fe1—C16—C17	119.3 (5)
C17—Fe1—C10—C12	119.0 (5)	C5—Fe1—C16—C17	160.8 (7)
C11—Fe1—C10—C12	−167.2 (3)	C11—Fe1—C16—C17	−80.9 (4)
C13—Fe1—C10—C12	−52.1 (6)	C13—Fe1—C16—C17	−164.4 (3)
C14—Fe1—C10—C12	162.5 (7)	C14—Fe1—C16—C17	−123.3 (3)
C5—C9—C11—C14	0.3 (5)	C12—C10—C17—C16	0.0 (6)
C8—C9—C11—C14	−177.6 (4)	Fe1—C10—C17—C16	59.2 (4)
Fe1—C9—C11—C14	−59.5 (3)	C12—C10—C17—Fe1	−59.2 (3)
C5—C9—C11—Fe1	59.8 (3)	C15—C16—C17—C10	0.5 (6)
C8—C9—C11—Fe1	−118.1 (4)	Fe1—C16—C17—C10	−59.3 (4)
C12—Fe1—C11—C14	164.1 (7)	C15—C16—C17—Fe1	59.8 (4)
C9—Fe1—C11—C14	119.4 (4)	C12—Fe1—C17—C10	38.0 (3)
C16—Fe1—C11—C14	−83.1 (4)	C9—Fe1—C17—C10	−82.5 (3)
C10—Fe1—C11—C14	−166.1 (3)	C16—Fe1—C17—C10	118.6 (5)
C15—Fe1—C11—C14	−51.4 (6)	C15—Fe1—C17—C10	81.3 (3)
C5—Fe1—C11—C14	81.6 (3)	C5—Fe1—C17—C10	−49.3 (6)
C17—Fe1—C11—C14	−125.4 (3)	C11—Fe1—C17—C10	−124.8 (3)
C13—Fe1—C11—C14	37.9 (3)	C13—Fe1—C17—C10	167.2 (6)
C12—Fe1—C11—C9	44.7 (8)	C14—Fe1—C17—C10	−164.9 (3)
C16—Fe1—C11—C9	157.5 (3)	C12—Fe1—C17—C16	−80.6 (4)
C10—Fe1—C11—C9	74.5 (4)	C9—Fe1—C17—C16	158.9 (3)
C15—Fe1—C11—C9	−170.8 (5)	C10—Fe1—C17—C16	−118.6 (5)
C5—Fe1—C11—C9	−37.8 (3)	C15—Fe1—C17—C16	−37.3 (3)
C17—Fe1—C11—C9	115.2 (3)	C5—Fe1—C17—C16	−167.9 (4)
C13—Fe1—C11—C9	−81.5 (3)	C11—Fe1—C17—C16	116.6 (4)
C14—Fe1—C11—C9	−119.4 (4)	C13—Fe1—C17—C16	48.6 (9)
C17—C10—C12—C15	−0.5 (6)	C14—Fe1—C17—C16	76.5 (4)
Fe1—C10—C12—C15	−60.2 (4)	C20—N3—C18—C19	−1.2 (7)
C17—C10—C12—Fe1	59.7 (3)	Co1—N3—C18—C19	174.3 (4)
C9—Fe1—C12—C15	−167.9 (3)	C20—N3—C18—C21^i^	178.7 (4)
C16—Fe1—C12—C15	37.2 (3)	Co1—N3—C18—C21^i^	−5.8 (5)
C10—Fe1—C12—C15	119.0 (5)	C23—C19—C18—N3	1.1 (8)
C5—Fe1—C12—C15	−126.0 (3)	C23—C19—C18—C21^i^	−178.8 (5)
C17—Fe1—C12—C15	81.3 (4)	C18—N3—C20—C22	0.4 (7)
C11—Fe1—C12—C15	157.5 (7)	Co1—N3—C20—C22	−174.5 (4)
C13—Fe1—C12—C15	−83.6 (4)	C23—C22—C20—N3	0.5 (8)
C14—Fe1—C12—C15	−48.0 (7)	C20—C22—C23—C19	−0.6 (8)
C9—Fe1—C12—C10	73.1 (4)	C18—C19—C23—C22	−0.2 (8)
C16—Fe1—C12—C10	−81.8 (3)	Co1—O6—C21—C18^i^	34.6 (5)

Symmetry codes: (i) −*x*, −*y*, −*z*.

### Hydrogen-bond geometry (Å, °)

**Table:**

*D*—H···*A*	*D*—H	H···*A*	*D*···*A*	*D*—H···*A*
O6—H6···O4	0.82	1.70	2.516 (5)	176
